# The Rise of Transradial Artery Access for Percutaneous Coronary Intervention in Patients with Acute Coronary Syndromes in Australia

**DOI:** 10.1155/2020/4397697

**Published:** 2020-11-27

**Authors:** Ryan James Ocsan, Ata Doost, Paul Marley, Ahmad Farshid

**Affiliations:** ^1^College of Health and Medicine, The Australian National University, Canberra, ACT, Australia; ^2^Department of Cardiology, Fiona Stanley Hospital, Murdoch, WA, Australia; ^3^Department of Cardiology, The Canberra Hospital, Canberra, ACT, Australia

## Abstract

**Objectives:**

The aim of this study was to evaluate the outcomes of acute coronary syndrome (ACS) patients undergoing percutaneous coronary intervention (PCI) via transradial artery access (TRA) or transfemoral artery access (TFA).

**Background:**

Over the last decade, evidence for the benefit of TRA for PCI has grown, leading to a steady uptake of TRA around the world. Despite this, the topic remains controversial with contrary evidence to suggest no significant benefit over TFA.

**Methods:**

A retrospective study of consecutive ACS patients from 2011 to 2017 who underwent PCI via TRA or TFA. The primary outcome was Major Adverse Cardiovascular Events (MACE), a composite of death, myocardial infarction (MI), target lesion revascularisation (TLR), or coronary artery bypass graft surgery (CABG) at 12 months. Secondary outcomes included Bleeding Academic Research Consortium (BARC) bleeding events scored 2 or higher, haematoma formation, and stent thrombosis, in addition to all individual components of MACE.

**Results:**

We treated 3624 patients (77% male), with PCI via TFA (*n* = 2391) or TRA (*n* = 1233). Transradial artery access was associated with a reduction in mortality (3% vs 6.3%; *p* < 0.0001), MI (1.8% vs 3.9%; *p*=0.0004), CABG (0.6% vs 1.5%; *p*=0.0205), TLR (1% vs 2.9%; *p* < 0.0001), large haematoma (0.4% vs 1.8%; *p*=0.0003), BARC 2 (0.2% vs 1.1%; *p*=0.0029), and BARC 3 events (0.4% vs 1.0%; *p*=0.0426). On multivariate Cox regression analysis, TFA, age ≥ 75, prior PCI, use of bare metal stents, cardiogenic shock, cardiac arrest, and multivessel coronary artery disease were associated with an increased risk of MACE.

**Conclusion:**

Despite the limitations secondary to the observational nature of our study and multiple confounders, our results are in line with results of major trials and, as such, we feel that our results support the use of TRA as the preferred access site in patients undergoing PCI for ACS to improve patient outcomes.

## 1. Introduction

Percutaneous coronary intervention (PCI) remains the definitive treatment for patients with acute coronary syndromes (ACS) [1]. In this setting, PCI is associated with both ischaemic and bleeding complications and the risk of haemorrhage from the arterial access site is amplified by concurrent administration of antiplatelet and anticoagulant drugs. Both major and minor bleeding events post-PCI are associated with worse outcomes, as they may trigger significant haemodynamic alterations, a need for blood transfusion, or an early cessation of antiplatelet therapy, which are all associated with increased cardiovascular events and mortality [2, 3].

There are multiple risk factors for post-PCI bleeding [4], including arterial access site. The radial artery is smaller and more superficial than the femoral artery, making haemostatic management more predictable. Caveats to transradial artery access (TRA) include the initial operator learning curve and experience [5, 6], age-related decline in vessel integrity [7], higher fluoroscopy times and thus, higher radiation exposure compared to transfemoral artery access (TFA) [8, 9], and uncommon radial-specific complications such as radial artery occlusion or perforation [9, 10]. The MATRIX trial of TRA versus TFA in ACS demonstrated a significant reduction in Major Adverse Cardiovascular Events (MACE) and bleeding with TRA [11], whereas the RIVAL trial did not show a difference in MACE or major bleeding events [12].

Hence, the role of arterial access choice in the reduction of MACE remains controversial. On balance, TRA has been increasingly found to be beneficial in improving outcomes particularly in high-risk groups (e.g., elderly, females, extremes of body mass index (BMI), and significant comorbidities) [7, 10, 13, 14]. Preference for TRA dominates in the United Kingdom and New Zealand [15], but adoption in the United States (US) [16] and Australia (with significant interstate variation) [15] has been slower, yet steadily increasing.

The results of randomised control trials (RCT) may not reflect the situation in real-world populations due to stringent selection criteria, under-representation of high-risk groups, exclusive study populations of either ST-segment elevation myocardial infarction (STEMI) [17, 18], or non-ST-segment elevation myocardial infarction (NSTEMI) [19, 20] and variable definitions of recordable bleeding events [11, 15, 17, 19, 21]. Our aim was to document the dramatic trend in adoption of TRA at our institution and determine if there were any differences in the occurrence of MACE and bleeding events using TRA or TFA in consecutive ACS patients undergoing PCI.

## 2. Materials and Methods

### 2.1. Study Setting

A retrospective analysis was conducted of the PCI registry at our tertiary referral centre that serves a population of approximately 700,000. Percutaneous coronary intervention is provided 24 hours a day for the management of STEMI patients and on-site cardiothoracic surgery is available. In addition to patients presenting with ACS to our Emergency Department, patients were also transferred urgently or semiurgently from several non-PCI centres ranging in distance from 15 to 200 km.

The analysis was conducted on patients admitted between January 2011 and December 2017 with a 12-month follow-up. The study was approved by the Research Ethics Committee as an ongoing clinical audit. All patients with ACS who subsequently underwent PCI were included. Diagnosis was made based on clinical presentation, electrocardiogram findings and cardiac biomarkers. Patients with stable angina and patients who died before the start of the procedure were excluded from this study.

The interventional procedure was conducted according to standard techniques. Patients were treated with aspirin (300 mg) and a P2Y_12_ receptor inhibitor (clopidogrel, prasugrel, or ticagrelor) prior to arrival at the catheterisation laboratory except in STEMI patients who were treated at the catheterisation laboratory. New P2Y_12_ inhibitors (prasugrel/ticagrelor) were available for use at our institution since 2011. Unfractionated heparin was given at the catheterisation laboratory and bivalirudin was not used. Percutaneous coronary intervention was performed by one of six operators via TRA or TFA at the operator's discretion. The TRA program at our institution began in 2012. Vascular closure devices (VCD) were used with TFA when clinically feasible, and TRA haemostasis was achieved using the TR Band (Terumo Corporation).

Patients' demographics, procedure details, and in-hospital complications were prospectively collected by research officers and entered into the PCI registry. Follow-up was carried out routinely at 12 months by letter, phone call, contact with the patients' primary doctor, and review of medical records, as previously described [22].

### 2.2. Definitions and Outcomes

Acute coronary syndrome was diagnosed as per the 4^th^ Universal Definition of Myocardial Infarction [23]. The primary outcome was MACE (a composite of death, myocardial infarction (MI), target lesion revascularisation (TLR), or coronary artery bypass graft surgery (CABG)) at 12 months. Target lesion revascularisation is defined as any repeat percutaneous intervention of the target lesion or bypass surgery of the target vessel performed for restenosis or other target lesion-related complications. Secondary outcomes included Bleeding Academic Research Consortium (BARC) bleeding events which scored 2 or higher [24], haematoma formation, and stent thrombosis, in addition to all individual components of MACE. Haematoma was defined as a swelling secondary to subcutaneous bleeding requiring medical intervention (i.e., BARC bleeding type 2). Stent thrombosis was defined as definite stent thrombosis according to the Academic Research Consortium criteria [24].

### 2.3. Statistical Analysis

Data were reported as numbers and percentages for categorical variables and means and standard deviations for continuous variables. Statistical analysis was performed using the Statistical Package for Social Sciences (Build 1.0.0.642, Version 25) (IBM, New York, USA). Categorical data were compared using chi-squared estimates and continuous data were compared using an unpaired Student's *t*-test. Multivariate Cox proportional hazards analysis was performed to identify predictors of MACE at 12 months. A forward likelihood ratio method was used to enter variables into the regression model including age ≥ 75, gender, cardiovascular risk factors, STEMI presentation, access site, multivessel coronary disease, prior PCI or CABG, use of P2Y_12_ receptor inhibitors, use of drug-eluting (DES) or bare metal stents (BMS), cardiogenic shock, and cardiac arrest.

## 3. Results

Between January 2011 and December 2017, 3624 patients with ACS were treated with PCI either via TFA (*n* = 2391) or TRA (*n* = 1233). Analysis of demographic data (Table 1) demonstrated that patients in the TRA group were younger (63.2 ± 12.1 years vs 65.5 ± 12.5 years; *p* < 0.0001) and had a lower percentage of females (20.1% vs 24.6%; *p*=0.0019) compared to the TFA group. Patients in the TRA group were more likely to be current smokers (26.8% vs 21.8%; *p*=0.0011), have a family history of cardiovascular disease (31.7% vs 27.0%; *p*=0.0032), and a higher BMI (29.0 vs 28.4; *p*=0.0048).

**Figure 1:**
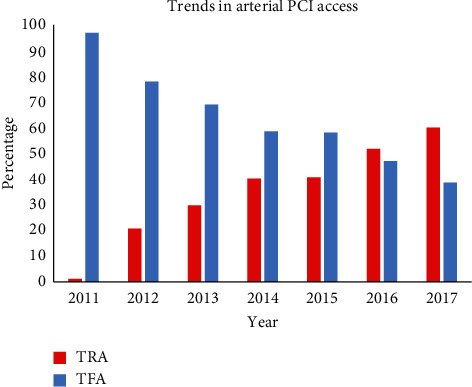
Trends in percutaneous coronary intervention (PCI) access site. TRA = transradial artery access. TFA = transfemoral artery access.

Procedural variables for each group are demonstrated in Table 2. Since the introduction of the TRA program in 2012, we have observed a steady rise in TRA from 1.8% in 2011 to 60.8% in 2017 (Figure 1). There was a higher prevalence of patients in the TFA group who experienced cardiogenic shock (5.1% vs 3.0%; *p*=0.0027) or cardiac arrest (2.8% vs 1.3%; *p*=0.0033), had a prior history of CABG (11.5% vs 2.7%; *p* < 0.0001), or PCI (24.8% vs 20.8%; *p*=0.0093). Additionally, there was higher use of glycoprotein IIb/IIIa inhibitors (GPI) (11.8% versus 4.3%, *p* < 0.0001), BMS (44.2% vs 23.4%, *p* < 0.0001), and intra-aortic balloon pumps (1.09% vs 0.08%; *p* < 0.0001) in the TFA group. Procedural success rates were comparable between groups but statistically higher with TRA. In addition, a greater use of new P2Y_12_ inhibitors (25.8% vs 22.6%; *p*=0.03) and smaller volumes of contrast (133.6 mL vs 144.2 mL; *p* < 0.0001) were observed in the TRA group.

Drug-eluting stents were used more frequently in the TRA group (68.7% vs 47.1%; *p* < 0.0001) and in approximately 8% of cases in each group stents were not used. These included unsuccessful procedures, cases of stent restenosis or thrombosis, treating small branches, and some bifurcation lesions and where a stent could not be deployed (e.g., vessel tortuosity and extensive calcification). Vascular closure devices were used in 57.5% of TFA patients and TR bands were used in all TRA patients for haemostasis.

Univariate analysis demonstrated better clinical outcomes with TRA compared to TFA (Table 3). A significant reduction in mortality at 12 months (3.0% vs 6.3%; *p* < 0.0001) was observed. In addition, the TRA group demonstrated lower rates of MI (1.8% vs 3.9%; *p*=0.0004), CABG (0.6% vs 1.5%; *p*=0.0205), TLR (1.0% vs 2.9%; *p* < 0.0001), large haematoma (0.4% vs 1.8%; *p*=0.0003), BARC 2 (0.24% vs 1.09%; *p*=0.0029), and BARC 3 bleeding events (0.41% vs 1.0%; *p*=0.0426). During this study, there was only 1 reported case of nonaccess site bleeding in the radial group related to a gastric bleed. Bleeding events that scored 4 or 5 were not observed in our study.

Multivariate analysis (Table 4) demonstrated that TFA was an independent predictor of MACE at 12 months (RR = 1.8; CI = 1.33–2.48; *p* < 0.0001). Other independent predictors of MACE included cardiogenic shock, cardiac arrest, age ≥ 75, multivessel coronary disease, prior PCI, and use of BMS.

## 4. Discussion

The publication of the major RCTs of TRA versus TFA in ACS [11, 12, 17, 18] has been accompanied by a steady increase in the adoption of TRA in many institutions [15, 16], including ours. Transradial artery access PCI for ACS at our institution steadily increased to over 60% of cases in a period of six years, representing the evolution of a major trend in interventional cardiology. This study represents our early experience with TRA but has already demonstrated that TRA was an independent predictor of reduced MACE at 12 months in patients with ACS. Bleeding Academic Research Consortium bleeding events that scored 2 or 3 were also significantly lower with TRA. Our results align with the MATRIX trial, the largest trial to date, which randomised 8404 patients with ACS to either TRA PCI or TFA PCI, demonstrating a significantly lower rate of MACE and bleeding with TRA [11]. A meta-analysis of 17 RCTs including the major RCTs to date also reflects this [25].

Overall results from RCTs suggest that TRA is associated with a lower risk of mortality in ACS but not in patients with stable ischaemic heart disease [10]. The RIVAL study did not find a significant difference in mortality between TRA and TFA, but mortality was significantly lower in the prespecified STEMI subgroup [12]. The MATRIX [11] and RIFLE-STEACS [17] trials found a lower mortality rate with TRA compared with TFA. The weighted mortality rate in patients with ACS was also demonstrated to be lower in TRA (2.7% vs 3.7%; *p* < 0.05) in a recent meta-analysis [10]. This translates to 10 fewer deaths for every 1000 patients with ACS undergoing TRA PCI.

The reasons behind a lower incidence of MACE with TRA remain unclear and likely multifactorial. One reason may be due to a reduction in bleeding events. Post-PCI bleeding is significant due to its association with worse clinical outcomes. Patients with ACS are generally treated with potent anticoagulant and antiplatelet agents and will have a higher risk of access site and nonaccess site bleeding compared with stable patients. Major bleeding events such as gastrointestinal or intracranial haemorrhage may necessitate interruption of antithrombotic medications, increasing the risk of stent thrombosis or other thrombotic events. Results from the one-year outcomes of the PRAGUE-18 study demonstrated that premature discontinuation of antiplatelet therapy was associated with significantly higher adverse events [26]. Additionally, blood transfusions used in the management of major bleeding have been linked with adverse short-term and long-term mortality [4].

Earlier mobility associated with TRA PCI may also drive a lower incidence of MACE. Earlier mobility and ultimately earlier discharge reduces the risk of venous thromboembolism and hospital-acquired complications [10]. In the same vein, lower rates of acute kidney injury with TRA result in shorter admissions and reduce the risk of chronic kidney disease [27].

Our study highlights several unique findings. Firstly, the reduction in MACE on univariate analysis was driven by several components including mortality, recurrent MI, TLR, and CABG. This is a novel finding given that, in a recent meta-analysis, a reduction in MACE with TRA was driven mainly by a reduction in mortality [28]. However, it is possible that lower rates of MI and TLR observed in our TRA group may be related to higher use of newer P2Y_12_ inhibitors and DES in this group. Secondly, our results arise from a centre wherein TRA was in its infancy and a significant learning curve existed as operators became familiar with the technique. We do not have accurate information on the rate of access site-switching; however, the estimated rate in the first two years was approximately 5%. Ultimately, this translates into a significant clinical benefit for TRA in a real-world consecutive cohort of ACS patients. Furthermore, we observed that improved clinical outcomes were demonstrated with TRA in the management of all subtypes of ACS and not limited to STEMI. It is likely that the relative benefit of TRA may be more pronounced when it is utilised in higher risk groups such as the elderly, females, extremes of BMI, and those with significant comorbidities [10].

Following the spawn of literature demonstrating the clinical benefits of TRA in ACS, TRA has been recommended as first-line PCI access route in the Australian [1], European [29], and US [10] guidelines. However, resistance to TRA persists. It has been suggested that better results obtained by TRA in the MATRIX trial were only apparent in high volume TRA centres, whose operators may have a perceived lower proficiency with TFA [6]. Our study represents encouraging results of an institution in transition from TFA to TRA (TRA = 2% in 2011 to 60% in 2017), with operators who were already skilled in TFA, gaining experience with TRA through the course of this study.

One of the caveats of the MATRIX trial was the use of GPIs [6], which is associated with increased bleeding and mortality [30]. In line with a global trend, our use of GPIs was declining during the period of this study. Overall usage of GPIs was 4.3% with TRA and 11.8% with TFA. We adjusted for the use of GPIs in our multivariate model and believe that a higher use of GPIs with TFA did not skew the observed benefits in the TRA group.

Other factors that may have contributed to higher risk of bleeding complications in older PCI trials include the use of larger sheaths and low usage of VCDs. Use of VCDs has been associated with a significant reduction in the risk of bleeding complications [31]. These were only used in 25.6% of TFA cases in the RIVAL study [12] and their use was not reported in the MATRIX study [11]. On the other hand, our operators rarely used sheaths larger than 6-French, and VCDs were utilised in 60% of TFA cases which is consistent with the frequency of usage of these devices in current studies. It is possible that higher usage of VCDs in our cohort may have reduced bleeding complications in the TFA group.

More recently, the SAFARI-STEMI trial failed to demonstrate any significant difference in 30-day mortality or bleeding complications between TRA and TFA primary PCI for STEMI patients [32]. Factors which may have reduced bleeding risk in SAFARI-STEMI included exclusion of postlysis and anticoagulated patients, predominant use of bivalirudin instead of heparin, avoidance of large femoral sheaths, and maximising the use of VCDs. Recruitment to the study was difficult and slow, eventually leading to premature cessation of the trial due to futility. Under these circumstances, it is likely that the randomised cohort represented a low-risk STEMI population, as operators may have been reluctant to randomise high bleeding risk patients (e.g., elderly or frail patients). Comparing TRA and TFA groups, the mean age was 61.6 versus 62.0; Killip Class II–IV demonstrated in 7% versus 6.7%, and 30-day mortality was 1.5% versus 1.3% (*p*=0.69%). If appropriate steps are taken to reduce bleeding risk as was done in the SAFARI-STEMI trial, the choice of vascular access does not significantly impact clinical outcomes in a relatively low-risk population of STEMI patients. While this notion is intuitive, the caveat is that certain components of the trial such as their exclusion criteria are not representative of routine day-to-day interventional practice, making it unlikely that their results can be translated into a real-world setting.

### 4.1. Limitations

The nonrandomised study design does not allow for the control of confounders between TRA and TFA groups. Interventionists may have favoured the use of TFA in the early part of their learning curve for STEMI or CABG patients, those with cardiac arrest or cardiogenic shock, elderly patients, and females. Use of DES was rapidly increasing during our study period of 2011–2017 in parallel with the rise in TRA and this resulted in greater overall usage of DES in the TRA cohort. However, we adjusted for all these variables in our multivariate model and still found a lower incidence of MACE with TRA. We acknowledge that due to the observational nature of the study and presence of multiple confounders, it is difficult to draw any firm conclusions regarding the effect of TRA on one-year MACE. This study spans over a decade and it is possible that PCI results naturally improved over time due to advancements in stent technology and antiplatelet agents, which may, in part, favour the outcomes in the TRA group. The data presented in this study were representative of our institution's clinical practice and may not be generalisable to other PCI centres. Finally, the retrospective study design prevents the capacity to audit the quality of patient data entered. However, data were collected at the time of the procedure by experienced technicians and are therefore likely to be accurate.

## 5. Conclusion

Transradial artery access was found to be an independent predictor of lower MACE and bleeding events at 12 months in consecutive patients with ACS treated with PCI. However, due to the observational nature of our study and the presence of multiple confounders, we caution against a definitive conclusion. Our findings from a real-world setting with consecutive patients and no exclusion criteria in an institution transitioning to TRA are in line with results of major RCTs. As such, we feel that our results support the use of TRA as the preferred access site in patients undergoing PCI for ACS to improve patient outcomes.

## Figures and Tables

**Table 1:** Patient demographics.

	TFA group	TRA group	*p* value
Overall population	2391 (66.0%)	1233 (34.0%)	

Age (years), mean + SD	65.5 ± 12.5	63.2 ± 12.1	<0.0001^*∗*^
Female, *n* (%)	585 (24.6%)	246 (20.1%)	0.0019^*∗*^
Diabetes, *n* (%)	547 (22.9%)	254 (20.6%)	0.1183
Diabetes treated with insulin	118 (4.9%)	61 (5.0%)	1.0000
Hypertension, *n* (%)	1331 (55.7%)	664 (53.9%)	0.3068
Smoker, *n* (%)	522 (21.8%)	330 (26.8%)	0.0011^*∗*^
Ex-smoker, *n* (%)	692 (28.9%)	380 (30.8%)	0.2492
Hypercholesterolaemia, *n* (%)	1053 (44.0%)	537 (43.6%)	0.8047
Family history of CVD, *n* (%)	646 (27.0%)	391 (31.7%)	0.0032^*∗*^
Body Mass index, mean + SD	28.4 ± 5.1	29 ± 5.7	0.0048^*∗*^
eGFR, mean + SD	74.9 ± 19.2	78.1 ± 16.1	0.007^*∗*^

Statistically significant (*p* < 0.05). TFA = transfemoral artery access. TRA = transradial artery access. SD = standard deviation. CVD = cardiovascular disease. eGFR = estimated glomerular filtration rate.

**Table 2:** Procedural variables.

	TFA group	TRA group	*p* value
Overall population	2391 (66.0%)	1233 (34.0%)	

Prior PCI, *n* (%)	572 (24.8%)	252 (20.8%)	0.0093^*∗*^
Prior CABG, *n* (%)	268 (11.5%)	32 (2.7%)	<0.0001^*∗*^
GPI use	281 (11.8%)	53 (4.3%)	<0.0001^*∗*^
Cardiogenic shock	123 (5.1%)	37 (3.0%)	0.0027^*∗*^
Cardiac arrest	68 (2.8%)	16 (1.3%)	0.0033^*∗*^
Indication			<0.0001^*∗*^
NSTEMI	890 (38.8%)	577 (47.3%)	
STEMI	1113 (48.6%)	489 (40.1%)	
Unstable angina	289 (12.6%)	154 (12.6%)	
Number of diseased vessels			0.5
1	1203 (51.4%)	638 (52.6%)	
2	659 (28.2%)	389 (32.1%)	
3	479 (20.5%)	185 (15.3%)	
Diseased artery segment			<0.0001^*∗*^
Graft	89 (3.7%)	11 (0.9%)	
Left main	44 (1.8%)	8 (0.7%)	
LAD/Diagonal	937 (39.3%)	480 (38.9%)	
LCx	550 (23.0%)	298 (24.2%)	
RCA	767 (32.1%)	436 (35.4%)	
Stent type			<0.0001^*∗*^
Balloon only	210 (8.8%)	98 (8.0%)	
BMS	1056 (44.2%)	288 (23.4%)	
DES	1125 (47.1%)	847 (68.7%)	
B2/C coronary lesion type	1786 (77.4%)	876 (73.2%)	0.0076^*∗*^
Prasugrel/ticagrelor use, *n* (%)	540 (22.6%)	318 (25.8%)	0.0321^*∗*^
Procedural success	2310 (96.6%)	1207 (97.9%)	0.0275^*∗*^
Vascular closure device use	1375 (57.5%)	N/A	<0.0001^*∗*^
Contrast volume, *mL*	144.2	133.6	<0.0001^*∗*^
Intra-aortic balloon pump use	26 (1.1%)	1 (0.9%)	<0.0001^*∗*^
TIMI flow			0.058
TIMI 0	42 (1.8%)	11 (0.9%)	
TIMI I	13 (0.6%)	7 (0.6%)	
TIMI II	65 (2.8%)	23 (1.9%)	
TIMI III	2208 (94.9%)	1166 (96.6%)	
Stent thrombosis			
Early (0–30 days)	9 (56.3%)	5 (71.4%)	0.487
Late (30–365 days)	6 (37.5%)	2 (28.6%)	0.676
Very late (>365 days)	1 (6.25%)	0 (0%)	0.388

Statistically significant (*p* < 0.05). TFA = transfemoral artery access. TRA = transradial artery access. PCI =  percutaneous coronary intervention. CABG =  coronary artery bypass graft. GPI = glycoprotein IIb/IIIa inhibitor. STEMI  =  ST-segment elevation myocardial infarction. NSTEMI = non-ST-segment elevation myocardial infarction. LAD = left anterior descending. LCx = left circumflex. RCA = right coronary artery. BMS = bare metal stent. DES = drug-eluting stent. TIMI = thrombolysis in myocardial infarction.

**Table 3:** Unadjusted analysis of procedural outcomes at 12 months.

	TFA group	TRA group	*p* value	Likelihood ratio
Death	150 (6.3%)	37 (3.0%)	<0.0001^*∗*^	19.4
Stent thrombosis	16 (0.7%)	7 (0.6%)	0.82	0.135
Myocardial infarction	93 (3.9%)	22 (1.8%)	0.0004^*∗*^	12.9
Target lesion revascularisation	69 (2.9%)	12 (1.0%)	<0.0001^*∗*^	15.6
CABG	35 (1.5%)	7 (0.6%)	0.0205^*∗*^	6.4
Haematoma	42 (1.8%)	5 (0.4%)	0.0003^*∗*^	14
BARC 2 bleeding events	26 (1.1%)	3 (0.2%)	0.0029^*∗*^	8.9
BARC 3 bleeding events	24 (1.00%)	5 (0.4%)	0.0426^*∗*^	4.1

Statistically significant (*p* < 0.05). TFA = transfemoral artery access. TRA = transradial artery access. CABG  =  coronary artery bypass graft. BARC  =  Bleeding Academic Research Consortium.

**Table 4:** Multivariate analysis of independent predictors of MACE at 12 months (Cox proportional hazard).

	Risk ratio	95% confidence interval	*p* value
Age ≥ 75	1.9	1.49–2.49	<0.0001^*∗*^
Female versus male	1.1	0.80–1.40	0.66
TFA versus TRA	1.8	1.33–2.48	<0.0001^*∗*^
Prior PCI	1.6	1.19–2.04	0.0013^*∗*^
Cardiogenic shock	3.3	2.19–4.88	<0.0001^*∗*^
Cardiac arrest	3.0	1.73–4.89	0.0002^*∗*^
DES versus BMS	0.6	0.47–0.77	<0.0001^*∗*^
Two-to-three vessel disease	1.7	1.33–2.17	<0.0001^*∗*^

Statistically significant (*p* < 0.05). TFA = transfemoral artery access. TRA = transradial artery access. PCI = percutaneous coronary intervention. DES = drug eluting stent. BMS = bare metal stent.

## Data Availability

In order to maintain patient privacy and confidentiality, data are not freely available for sharing.
